# A self-oblique exercise that activates the coordinated activity of abdominal and hip muscles–A pilot study

**DOI:** 10.1371/journal.pone.0255035

**Published:** 2021-08-12

**Authors:** Yuki Nakai, Masayuki Kawada, Takasuke Miyazaki, Sota Araki, Yasufumi Takeshita, Ryoji Kiyama

**Affiliations:** 1 Department of Mechanical Systems Engineering, Faculty of Engineering, Daiichi Institute of Technology, Kagoshima, Japan; 2 Department of Physical Therapy, School of Health Sciences, Faculty of Medicine, Kagoshima University, Kagoshima, Japan; 3 Doctoral Course, Graduate School of Health Sciences, Kagoshima University, Kagoshima, Japan; University of Innsbruck, AUSTRIA

## Abstract

The importance of an interaction between trunk stability muscles and hip muscle function has been suggested. However, reported exercises rarely act on the trunk and hip muscles simultaneously. Here, we devised an abdominal oblique and hip muscle exercise, the Self-oblique exercise (SOE). We examined whether SOE activated abdominal and hip muscles in the supine and half-kneeling positions, compared with abdominal crunch (AC) and plank exercises; and whether participants could modulate the exercise load. Participants were 20 healthy males with some sports experience such as football and baseball on average 10.5 ± 4.0 years. Participants applied self-pressure to their right thighs using the contralateral upper limb with 40% or 70% of the maximum force in Supine SOE and Half- kneeling SOE. The following abdominal and hip muscles were measured using surface electromyography: bilateral external obliques (EO), bilateral internal obliques (IO), right rectus abdominis, right gluteus medius (GMed), and right adductor longus (ADD). All evaluated muscle groups showed significant differences between exercises (p < 0.001). Supine SOE-70% showed 80.4% maximal voluntary contraction (MVC) for left EO (p < 0.017), 61.4% MVC for right IO (p < 0.027), 24.3% MVC for GMed (p < 0.002), and 42.4% MVC for ADD (p < 0.004); these were significantly greatest among all exercises. Muscle activity during Supine SOE-70% was greater than that during Supine SOE-40%. Similarly, Half-kneeling SOE-40% promoted abdominal and hip muscle exertion, and showed more significant activity in GMed (p < 0.006) and ADD (p < 0.001) than AC and plank. SOE could activate abdominal and hip muscles depends on the pressure applied by upper limb. Also, SOE allows participants to modulate the exercise load in a self-controlled step by step manner. Modulation of the exercise load is difficult in AC or plank compared to SOE, and AC or plank cannot obtain simultaneous oblique and hip muscle activity. SOE could be practiced anywhere, in various positions, without any tools.

## Introduction

Core stabilization exercises are often used for lower limb musculoskeletal training and injury prevention [1]. Core muscles, including the internal oblique muscles and transversus abdominis, play an important role in trunk stabilization [2, 3] because core muscles attach to the spine through the thoracolumbar fascia to increase spinal column stability [4]. However, there is increasing evidence that lumbar spine, pelvis, and lower limb muscles do not work in isolation, but that they are interrelated with each other. For example, the addition of contraction of deep abdominal muscles increased hip muscle activity during hip exercise [5]. Systematic reviews have shown the importance of trunk muscle function in preventing lower limb injury [6]. Pelvic instability can increase the load on lower limb muscles and joints through the kinematic chain, inducing distal lower limb injury [7]. Meanwhile, Individuals with lower back pain show various types of hip dysfunction, including reduced endurance and delayed activation of hip extension and abduction [8]. Poor trunk strength and control, as well as hip muscle function, are associated with risk factors for lower limb injury [9–11]. Although these findings indicate the importance of coordinated trunk and hip activity, few exercises simultaneously activate these muscles.

Exercises that facilitate coordinated activation of the trunk and hip muscles would be enhance performance, but previous studies have not provided an effective exercise. A few studies report that effective athletic training, such as the Copenhagen Adduction Exercise promotes simultaneous activation of the hip adductors and abdominal muscles [12–14]. However, these exercises could only be performed in a specific posture or required tools such as tubes or unstable plates, and therefore had a low level of convenience for exercise position and modulation of exercise load. Each individual has a different trunk and hip stability muscle strength, thus adequate exercise posture and load should depend on an individual’s characteristics. In addition, external loads may pose a risk of overload that can result in personal injury; therefore, in order to optimize the effect, exercises of the trunk and hip muscles should be independent, self-voluntary, and step-adjustable [15, 16].

Sports activities are usually performed in an anti-gravity position. Exercises specifically aimed at improving these movements should also be performed in an anti-gravity posture such as sitting or standing, but studies that report such exercises are scarce [17, 18]. Half-kneeling is an anti-gravity position and has previously been used as a test for core stability [19]. In previous studies, trunk stabilizing exercises with unilateral isometric hip rotation in the supine position effectively activated ipsilateral internal obliques (IO) [20], while unilateral dumbbell lifting in the sitting position increased contralateral external obliques (EO) muscle activity [21]. These findings suggest that the load on the unilateral limb effectively promotes activity of the oblique muscles to counter the rotational moment acting on the trunk. We devised the new self-oblique exercise (SOE) that can promote coordinated activation of the abdominal and hip muscles in a supine and half kneeling position. This trunk stabilization exercise could be promoted simultaneously with these muscles being activated by applying pressure to the thigh using the participants’ own contralateral upper limb in two positions. The exercise load of SOE is determined by self-applied pressure using the participants upper limb so that an appropriate load can be selected by each individual.

The purpose of this study was to examine abdominal and hip muscle activity during the newly developed exercise, and to compare this activity during two traditional trunk stabilization exercises, using surface electromyography (EMG). Developing step-by-step exercises might lead to the prescription of proper exercises based on the tolerance and physical performance of individuals. We hypothesized that SOE would simultaneously activate oblique muscles and hip adductor muscles in two positions, and that those activations would be regulated by the self-applied pressure.

## Materials and methods

### Design

This study was a cross-sectional within-subjects study. The Kagoshima University’s Ethics Committee on Epidemiological Studies approved this study (approval No. 170116 Epi ver. 1). We compared the muscle activity of core and hip muscles during our defined SOE and the traditional exercise abdominal crunch (AC) and plank using surface EMG. Prior to measurement, each subject practiced each exercise condition for 10 minutes with an experienced therapist. The order of the attempts was randomized using a random number table created in Microsoft Excel. Each exercise was performed under isometric contraction for 7 seconds and was repeated 3 times. To prevent muscle fatigue of each subject, a rest time of 30 seconds between attempts and a rest time of 2 minutes between attempt conditions were provided [22].

### Participants

Twenty physically active males participated in this study (24.6 ± 3.4 y, 1.71 ± 0.06 m, 63.0 ± 7.6 kg). All participants had some sports experience (10.5 ± 4.0 y). The sports experience was as follows: 6 people in football, 2 people each in baseball, basketball, volleyball, athletics, and kendo, and 1 person each in handball, tennis, badminton, and table tennis. Participants were excluded if they reported a history of any of the following within the last 6 months that could affect athletic performance: 1) lower limb injury, 2) lower back or lower limb joint surgery, 3) neurological disease. All participants were informed of the risks and benefits of the study before providing written informed consent. The individual in this manuscript has given written informed consent (as outlined in PLOS consent form) to publish these case details. Sample size was calculated using G * Power 3.1.9.2 according to a previous report comparing EO muscle activity during plank [23]. Power analysis showed that at least 15 participants were required to achieve a power of 0.90 at p < 0.01.

### Procedures

Self-applied pressure force was estimated by the handheld dynamometer. The analog output from the handheld dynamometer was transmitted to a computer at 1000 Hz through a 16-bit A/D converter (NI USB-3643, National Instruments, Austin, TX, USA). The pressure measured by a dynamometer was displayed by a projector on the ceiling or the wall in real time. Participants were instructed to adjust their pushing force to 40% or 70% of the maximum force for 7 seconds under visual feedback conditions during Supine SOE and Half-kneeling SOE.

Body hair was shaved from areas needed for electrode connection and the skin was washed with alcohol-soaked cotton to minimize skin impedance. After skin preparation, disposable electrodes (Blue Sensor M-00-S, Medicotest, Olstykke, Denmark) were placed along the axis of EO, IO, rectus abdominis (RA), gluteus medius (GMed), and adductor longus (ADD) with a 2 cm inter-electrode distance. The electrodes were placed above and below the rib margin for EO, this was at the lower margin of the eighth rib; 2 cm inferomedial to the superior anterior iliac spine for IO; approximately 3 cm outside the umbilicus for RA [24]; on the midpoint of the line connecting the ililac crest and the greater trochanter of the femur for GMed [25, 26]; and approximately 4 fingerbreadths distal to the pubic tubercle over the bulk of the adductor muscles for ADD [27]. The method used for measuring IO activity in this study was previously validated and compared with muscle thickness estimated by ultrasound imaging [20]. EMG signals were collected for muscle activity using Telemyo DTS system (Noraxon Inc., Scottsdale, AZ, USA). Frequency response was 10 to 500 Hz, variable input impedance was 100 MΩ or more, common mode rejection rate was 100 dB or more, sampling frequency 1500 Hz. Using Matlab 2017 (Mathworks Inc, Natick, Massachusetts, USA), the 5-second data during the 7-second EMG recorded in each exercise was filtered with a band-pass filter with a cutoff of 50–500 Hz and intergraded after full-wave rectification. Normalization of the integrated EMG was performed by the maximal EMG obtained during maximal voluntary contraction (MVC) according to the manual muscle test procedure and was taken as the percentage of MVC (% MVC) [28]. The mean % MVC values obtained from each exercise were compared and analyzed.

### Exercise conditions

Bilateral EO and bilateral IO, right RA, right GMed, and right adductor longus (ADD) muscle activity were measured using EMG during 5 exercises, including AC, plank, Supine SOE (40% and 70%), and Half-kneeling SOE-40%. The Supine SOE (40% and 70%), and Half-kneeling SOE-40% was defined as 40% or 70% of the maximum pushing force measured in the supine position in advance. Muscle activity during exercise was compared cross-sectionally.

**Fig 1:**
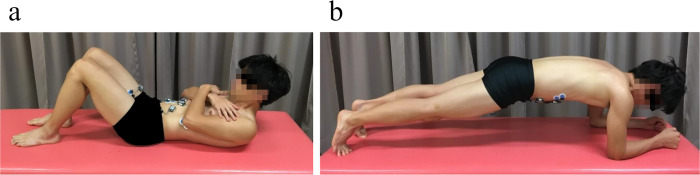
Traditional core stabilization exercise from lateral view. a) Abdominal Crunch, b) Plank.

AC was performed in the supine position. Participants folded both arms on their chest and bent their knees to 90°. They lifted their heads and shoulders until the shoulder blades cleared the bed [29] (Fig 1A). Plank was performed in the prone position. Participants placed their forearms on the bed, keeping their feet apart, and their spine and pelvis in a neutral position. Their elbows were spaced shoulder width just below the glenohumeral joint. They lifted their bodies with their forearms and toes and maintained a straight alignment [23] (Fig 1B).

**Fig 2:**
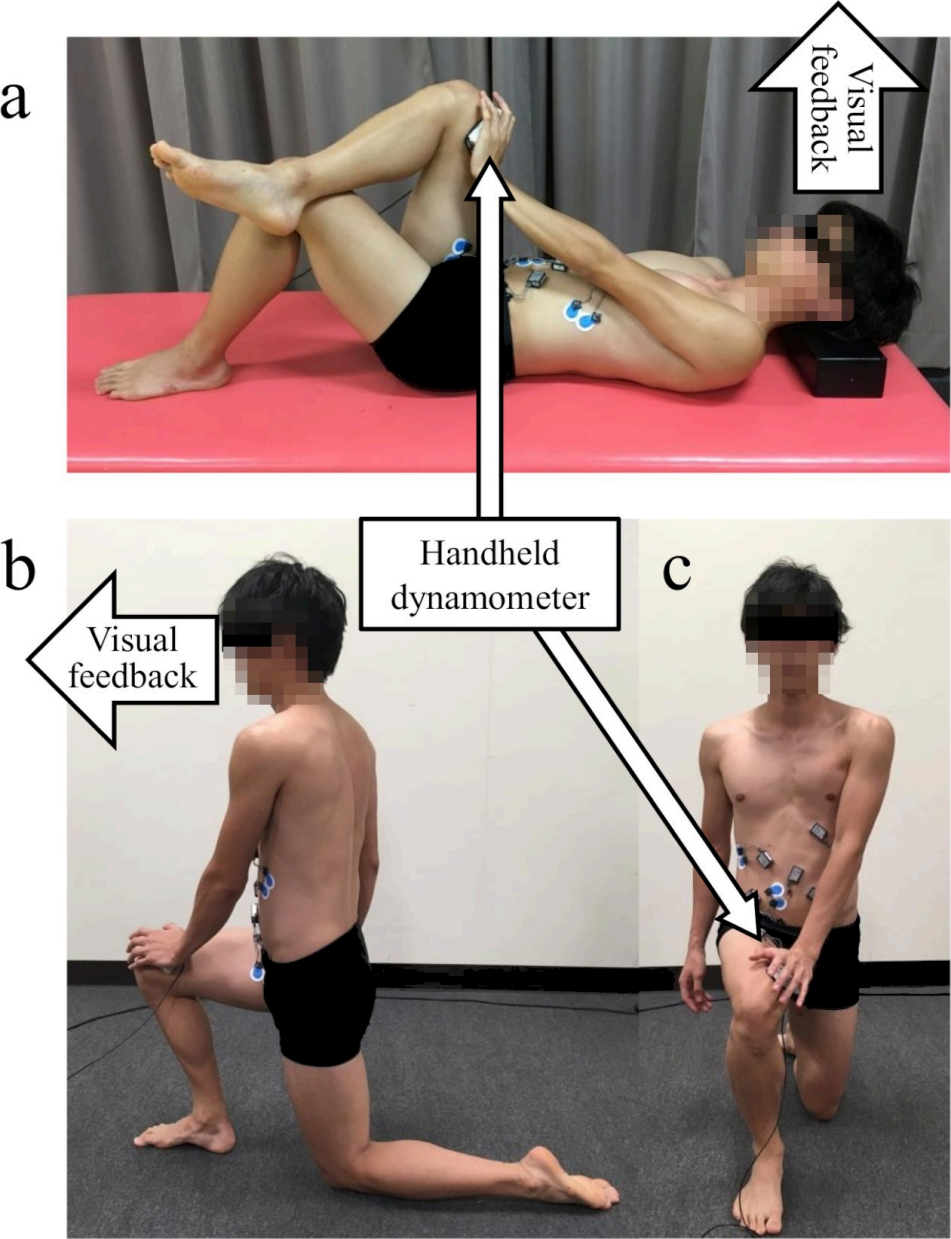
Stabilization exercise of core and hip muscles. a) Self-oblique exercise in a supine position from lateral view, b) The Half-kneeling Self-oblique exercise was performed with the right leg in front, from lateral view; c) from anterior view.

Supine SOE was performed in the supine position, bending the left knee 90° and placing the lateral malleolus of the right fibula on the left knee joint. Participants were asked to apply pressure to the medial condyle of the right femur with the dynamometer held in their left hand without raising their head (Fig 2A). They were instructed to maintain a neutral position of the spine and pelvis to eliminate compensatory movements [30]. Left external oblique (LEO) and right internal oblique (RIO) were activated to rotate the upper body to the right and to apply pressure to their right thigh. In addition, GMed and ADD were activated to oppose the external rotation and abduction forces of the right hip joint caused by the pressure applied by the left hand, in this exercise. We reported that in the supine position and 90° knee flexion, participants activated the internal oblique muscles and maintained a neutral position against unilateral hip abduction and abduction loads [20]. We assumed that if participants exerted pressure on the contralateral lower limbs with their contralateral upper limbs, they would gain activity in the hip adduction and internal rotation muscles as well as in the oblique muscles. Prior to the measurement of muscle activity, the maximum pressure from the left hand to the right thigh was measured by the handheld dynamometer. Self-applied pressure during Supine and Half-kneeling SOE was determined based on the maximum force. Participants pushed their thigh by 40% or 70% of the maximum force during Supine SOE. Half-kneeling SOE was performed with the right leg in front. The right hip and right knee were bent 90°, and the left knee was placed on the floor in a 90° bent position [31]. Participants applied pressure to their thigh using the left hand in a manner similar to Supine SOE, while the pelvis and spine were held in a neutral position. Participants applied pressure to their thigh at 40% of maximum force during Supine SOE (Fig 2B and 2C).

### Statistical analyses

The EMG reproducibility of the three trials, during all exercise conditions, was confirmed by an intra-class correlation coefficient (ICC_1,3_) based on the collected data, and was in the range of 0.87–0.99 (95% confidence interval; 0.73–0.99, p < 0.001). The normal distribution of the collected data was checked using the Shapiro-Wilk test. Differences in muscle activity for each exercise condition were tested using repeated measures analysis of variance when the data was normally distributed, and using the Friedman test when the data was not normally distributed. Effect sizes were calculated by eta-square for repeated measures analysis or Kendall’s W for Friedman test [32, 33]. As a post hoc test, Tukey’s test or Wilcoxon rank-sum test, using p-values adjusted by Holm’s method, was performed. Statistical analyses were performed using R-2.8.1 software (R Foundation for Statistical Computing, Vienna, Austria). The significance level was set at p < 0.05.

## Results

**Fig 3:**
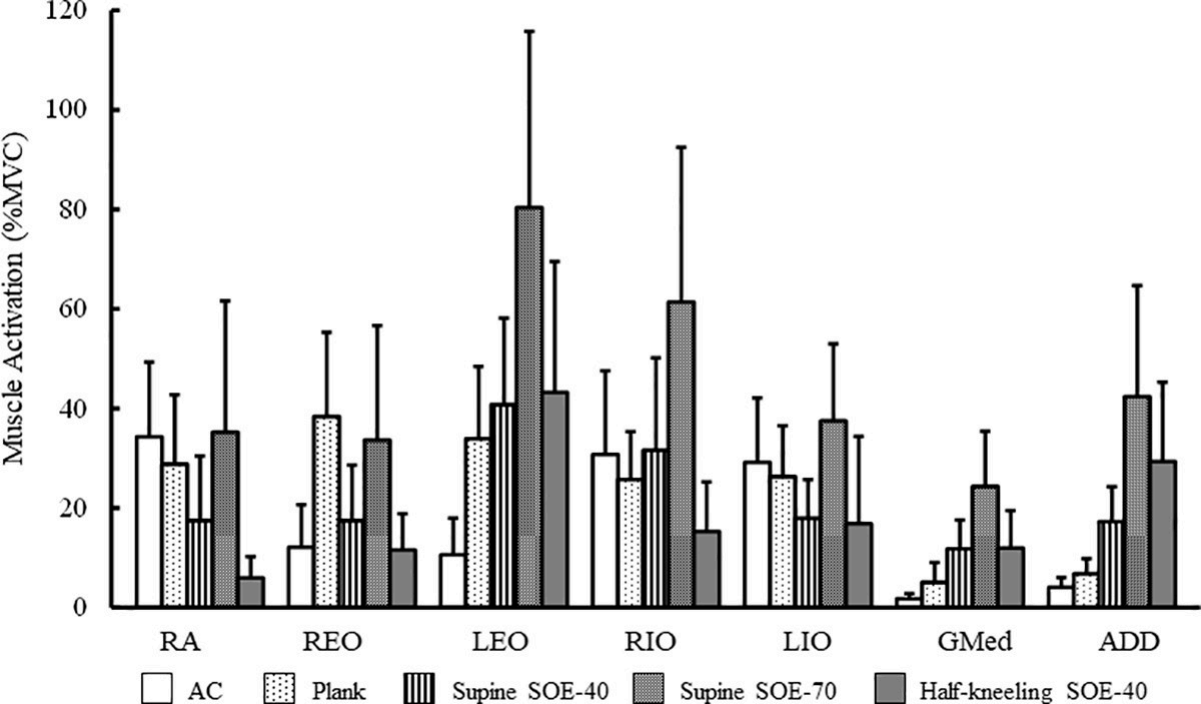
Comparison of muscle activity during the exercises for each muscle. Muscle activity showed the percentage of maximal voluntary contraction (% MVC; mean ± SD). Abbreviations: RA = rectus abdominis; REO = right external oblique; LEO = left external oblique; RIO = right internal oblique; LIO = left internal oblique; Gmed = gluteus medius; ADD = adductor longus. The significant differences between the exercises were listed in Table 1.

Since the Shapiro-Wilk test showed that at least one of the muscle activities during each exercise did not adopt on a normal distributed, all data were analyzed using Friedman test and Wilcoxon rank-sum test adjusted by Holm’s method. Muscle activity of all muscles showed significant differences among the 5 exercises (Fig 3; χ^2^ = 32.2–68.1, p < 0.001), and SOE simultaneously activated LEO, RIO, GMed, and ADD muscle activity as expected (S1 File). Effect sizes of LIO was shown to be medium (p < 0.001, Kendall’s W = 0.40), while other muscles showed large effect size (p < 0.001, Kendall’s W = 0.50–0.85). Supine SOE-40% showed 40.8 ± 17.4% MVC for LEO, 31.6 ± 18.6% MVC for RIO, and were equal to or higher than those of AC and plank (Table 1C and 1D). Similarly, Supine SOE-40% showed 11.8 ± 5.7% MVC for GMed and 17.3 ± 7.0% MVC for ADD, indicating a significantly higher level of activity than AC (Table 1F) and plank (Table 1G). Supine SOE-70% showed 80.4 ± 35.4% MVC for LEO (Table 1C), 61.4 ± 31.1% MVC for RIO (Table 1D), 24.3 ± 11.1% MVC for GMed, and 42.4 ± 22.3% MVC for ADD; these were significantly the highest figures among all exercises. Muscle activity during Half-kneeling SOE-40% was 43.3 ± 26.3% MVC for LEO, 12.0 ± 7.4% MVC for GMed, and 29.3 ± 15.9% MVC for ADD, showing an equal to or higher level of activity than that of AC and plank. Conversely, RIO activity during Half-kneeling SOE-40% was 15.3 ± 9.9% MVC, which was lower than that of AC and plank.

**Table 1:** Difference in %MVC muscle activity between exercise conditions.

		Plank	Supine	Supine	Half-kneeling
			SOE-40	SOE-70	SOE-40
A. Rectus Abdominis					
	AC (34.3 ± 15.0%MVC)	-5.5	-16.8**	0.9	-28.4**
	Plank (28.8 ± 13.9%MVC)		-11.4**	6.4	-22.9**
	Supine SOE-40 (17.5 ± 13.0%MVC)			17.8**	-11.5**
	Supine SOE-70 (35.2 ± 26.3%MVC)				-29.3**
	Half-kneeling SOE-40 (6.0 ± 4.3%MVC)				
B. Right External Oblique					
	AC (12.1 ± 8.5%MVC)	26.3**	5.3	21.5**	-0.5
	Plank (38.4 ± 16.9%MVC)		-21.0**	-4.8	-26.8**
	Supine SOE-40 (17.4 ± 11.2%MVC)			16.2**	-5.8**
	Supine SOE-70 (33.6 ± 23.0%MVC)				-22.0**
	Half-kneeling SOE-40 (11.6 ± 7.2%MVC)				
C. Left External Oblique					
	AC (10.6 ± 7.4%MVC)	23.3**	30.2**	69.8**	32.7**
	Plank (33.9 ± 14.5%MVC)		6.9	46.5**	9.4
	Supine SOE-40 (40.8 ± 17.4%MVC)			39.6**	2.5
	Supine SOE-70 (80.4 ± 35.4%MVC)				-37.1*
	Half-kneeling SOE-40 (43.3 ± 26.3%MVC)				
D. Right Internal Oblique					
	AC (30.8 ± 16.8%MVC)	-5.1	0.8	30.6*	-15.5**
	Plank (25.7 ± 9.6%MVC)		5.9	35.7**	-10.4*
	Supine SOE-40 (31.6 ± 18.6%MVC)			29.8**	-16.3**
	Supine SOE-70 (61.4 ± 31.1%MVC)				-46.1**
	Half-kneeling SOE-40 (15.3 ± 9.9%MVC)				
E. Left Internal Oblique					
	AC (29.2 ± 12.9%MVC)	-2.9	-11.3*	8.3	-12.4
	Plank (26.3 ± 10.2%MVC)		-8.4*	11.1*	-9.5
	Supine SOE-40 (17.9 ± 7.7%MVC)			19.6**	-1.1
	Supine SOE-70 (37.5 ± 15.5%MVC)				-20.7*
	Half-kneeling SOE-40 (16.8 ± 17.6%MVC)				
F. Gluteus Medius					
	AC (1.8 ± 0.9%MVC)	3.2**	10.0**	22.5**	10.1**
	Plank (5.1 ± 4.0%MVC)		6.8**	19.3**	6.9**
	Supine SOE-40 (11.8 ± 5.7%MVC)			12.5**	0.2
	Supine SOE-70 (24.3 ± 11.1%MVC)				-12.3**
	Half-kneeling SOE-40 (12.0 ± 7.4%MVC)				
G. Adductor Longus					
	AC (4.1 ± 2.0%MVC)	2.7**	13.2**	38.3**	25.2**
	Plank (6.8 ± 3.1%MVC)		10.5**	35.6**	22.6**
	Supine SOE-40 (17.3 ± 7.0%MVC)			25.1**	12.0**
	Supine SOE-70 (42.4 ± 22.3%MVC)				-13.0**
	Half-kneeling SOE-40 (29.3 ± 15.9%MVC)				

Data presented as mean ± standard deviation. Abbreviations: %MVC = percentage of maximal voluntary contraction; AC = abdominal crunch; SOE = self-oblique exercise.

** p < 0.01

* p < 0.05.

## Discussion

The purpose of this study was to investigate whether SOE, performed using self-applied pressure to the thigh with the contralateral upper limb, could simultaneously activate muscle activity in oblique and hip muscles in supine and half-kneeling positions. In addition, whether participants could modulate the exercise load step by step by themselves. The results showed that SOE elicited coordinated muscle activity of EO, IO, GMed, and ADD muscles more effectively than AC and plank. Participants were also able to adjust the exercise load on their own. These results suggest that SOE can gradually increase the load and simultaneously activate oblique and hip muscle activities in the supine and half-kneeling positions, in physically active males.

EO, IO, and RA activities under the plank condition measured in this study was medium, similarly to the values shown in a previous study [34], while hip muscle activities were low. Muscle activity under the AC condition was low for EO, medium for IO and RA, which was similar to previous studies [35, 36], while hip muscle activity was very low. Meanwhile, plank and AC promoted muscle activity in the abdominals, but not in the hip abductor and adductor muscles. Because AC and plank are sagittal plane exercises, they activate RA but not the GMed and ADD.

Abdominal and hip muscle activities in Supine SOE-40% were 41% for LEO, 32% for RIO, 12% for GMed, and 17% for ADD of MVC. The purpose of Supine SOE-40% was to activate muscle activity of LEO and RIO; activated muscle activity was comparable to those during plank and AC. Activated muscle activity during SOE-40% was also equivalent to GMed in Squats [37] and equivalent to ADD in Ball squeezes [38]. SOE was performed by self-applied pressure to the thigh using the contralateral upper limb. The pressure applied by the upper limb acted as an external abduction and external rotation force on the contralateral hip joint, and activated muscle activity relating to hip adduction and internal rotation. Simultaneously, reaction force acting from the thigh to the contralateral upper limb generated rotational force in the upper trunk, eliciting the oblique muscles. Therefore, SOE could promote coordinated muscle activity of abdominal and hip muscles, and could modulate the exercise load depending on the pressure applied by the upper limb.

Abdominal and hip muscle activities in Supine SOE-70% were 80% for LEO, 61% for RIO, 24% for GMed, and 42% for ADD of MVC. These greatly exceeded the muscle activity of 34% for LEO and 26% for RIO during plank. In addition, Supine SOE-70% was able to activate GMed muscle activity equivalent to banded side-stepping [39], and ADD significantly exceeded side-lying hip adduction [38] in a previous study. These results suggested that Supine SOE was able to regulate abdominal and hip muscle activities in a self-progressive manner.

Half-kneeling SOE-40% promoted activation of LEO and hip muscles, and muscle activity of LEO, GMed, and ADD was greater than during AC and plank, while muscle activity of RA, REO, and RIO was lower. A previous study suggests that the vertical position results in less lever arm of the body weight to the joints of the spine when compared to the horizontal position [14]. This kinetic characteristic of position would relate to the low activation of abdominal muscles excluding the LEO. In addition, postural instability during half-kneeling, unlike the supine position, might reduce muscle activation of the abdominal muscles. Trunk stability depends on the coordinated activation of core and hip muscles, and is a key factor in preventing lower back pain and lower limb injury during sporting activities. Thus, regardless of the stability, abdominal muscles need to be activated in various positions for performing lower limb function adequately. There are few self-exercises that promote the core and hip muscles in an anti-gravity position [1] and we recommend the Half-kneeling SOE for this purpose. In the present study, SOE simultaneously promoted the abdominal and hip muscles in the supine and half-kneeling positions. In addition, exercise load was able to be delicately regulated depending on individual status; therefore, SOE is a practical self-exercise for physically active males.

This study had some limitations. During EMG measurements, there was a risk of crosstalk between surrounding muscles [40]. All participants were healthy males without back pain, and results may not always be generalizable to other populations. Meanwhile, we analyzed cross-sectional muscle activation during SOE, thus the longitudinal effects of SOE should be confirmed in future studies.

## Conclusions

SOE activated concurrent oblique and hip muscle activity and exhibited greater oblique and hip activity compared to traditional AC and plank. In addition, SOE was able to modulate the exercise load in a self-controlled step by step manner. Similar activities were observed under the Half-kneeling SOE condition, which is a position in an anti-gravity condition. SOE could be performed anywhere without tools SOE would be tailored to the individual’s physical fitness, and may be practical in promoting oblique and hip muscle activity.

## Supporting information

S1 File Participant characteristics and EMG data. (XLSX)
